# Using Formative Research to Design a Behavior Change Strategy to Increase the Use of Improved Cookstoves in Peri-Urban Kampala, Uganda

**DOI:** 10.3390/ijerph10126920

**Published:** 2013-12-10

**Authors:** Stephanie L. Martin, Jennifer K. Arney, Lisa M. Mueller, Edward Kumakech, Fiona Walugembe, Emmanuel Mugisha

**Affiliations:** 1 PATH, 455 Massachusetts Avenue, NW, Suite 1000, Washington, DC 20001, USA; E-Mails: jarney@path.org (J.K.A.); lmueller@path.org (L.M.M.); 2 Division of Nutritional Sciences, Cornell University, 118 Savage Hall, Ithaca, NY 14853, USA; E-Mail: slm345@cornell.edu; 3 PATH, P.O. Box 24578, Kampala, Uganda; E-Mails: ekumakech@path.org (E.K.); fwalugembe@path.org (F.W.)

**Keywords:** household air pollution, indoor air pollution, improved cookstoves, behavioral change, formative research, qualitative research, health technology, theoretical framework, Uganda

## Abstract

Household air pollution from cooking with biomass fuels negatively impacts maternal and child health and the environment, and contributes to the global burden of disease. In Uganda, nearly 20,000 young children die of household air pollution-related pneumonia every year. Qualitative research was used to identify behavioral determinants related to the acquisition and use of improved cookstoves in peri-urban Uganda. Results were used to design a behavior change strategy for the introduction of a locally-fabricated top-lit updraft gasifier (TLUD) stove in Wakiso district. A theoretical framework—opportunity, ability, and motivation—was used to guide the research and behavior change strategy development. Participants consistently cited financial considerations as the most influential factor related to improved cookstove acquisition and use. In contrast, participants did not prioritize the potential health benefits of improved cookstoves. The theoretical framework, research methodology, and behavior change strategy design process can be useful for program planners and researchers interested in identifying behavioral determinants and designing and evaluating improved cookstove interventions.

## 1. Introduction

More than one-third of the world’s population burns biomass to cook and heat their homes [1]. Household air pollution from burning biomass and other solid fuels is responsible for an estimated 3.5 million deaths annually—accounting for 4.5 percent of the global burden of disease [2]. Women and children in low-resource countries are disproportionately affected [3], due to their high levels of exposures associated with cooking several hours each day with traditional stoves [4]. Women in low-income countries are least likely to be able to access cleaner fuels and cook stoves [5]. Among children less than five years old, household air pollution is the third leading risk factor for mortality [2]. Exposure to household air pollution from burning biomass is associated with increased risk of acute lower respiratory infections in children and chronic obstructive pulmonary disease in adult women [6,7,8]. Evidence points to links between household air pollution and cardiovascular disease, cataracts, tuberculosis, low birth weight, and impaired cognitive function in children [2,3,4,9]. In addition to health impacts, the burning of biomass for cooking and heating contributes to ambient air pollution [4,10], deforestation [11], and global climate change [11,12]. 

In Uganda, biomass serves as the main source of energy and, at 95 percent, its use is almost universal [13]. Solid fuel use in Uganda contributes to 5 percent of the national disease burden and nearly 20,000 young children die of household air pollution-related pneumonia every year [14]. Recent analysis of national survey data investigating the relationship between housing quality and occupant health in Uganda found that burning biomass for cooking was associated with morbidity more than any other physical housing attribute measured [15].

Within the past decade, there has been an increased international effort to address household air pollution, including the formation of the Global Alliance for Clean Cookstoves led by the United Nations [16]. Scaling-up the use of improved cookstoves is a central component of this effort. Improved cookstoves are cookstoves that are able to meet the household’s cooking needs, are durable and affordable, are acceptable to the household, consume less fuel than traditional stoves or a three-stone fire, and reduce household air pollution to levels that will improve health [17,18]. These stoves are more efficient than traditional and unimproved stoves because they require less fuel for cooking, less time for gathering fuel, and less time for cooking. These advantages have the potential to improve household health and finances, as well as benefit the local environment and reduce global climate change [19]. Although the efficacy of some improved cookstoves to reduce emissions in highly-controlled laboratory and field trials has been reported, there is a lack of evidence on the effectiveness of improved cookstove interventions in real-world settings [19,20]. At this point in time, improved cookstoves have largely failed to play an important role in reducing maternal and child deaths from burning biomass [21]. 

Formative research is a process that is used to better understand behavioral determinants in order to design and implement culturally appropriate and effective interventions and programs [22]. The objectives of this formative research were to document village health volunteers, community leaders, and relevant ministry staff perceptions of improved cookstoves, and identify the related barriers and facilitating factors community members perceive and experience. The data collected was used to inform a behavior change strategy to increase the acquisition and use of the TLUD stove specifically.

This paper describes the formative research methods and findings, and the approach for developing a targeted behavior change strategy based on the opportunity, ability, and motivation theoretical framework. The study focuses on low-income, peri-urban households in Wakiso District, Uganda, which is an important target audience given rapid urbanization rates in Uganda and the high levels of poverty in peri-urban areas. This study is relevant to other improved cookstove programs and will help in the design of larger behavior change strategies focused on scaling-up the acquisition and sustained use of improved cookstoves. Subsequent papers will describe the impact of the behavior change strategy on practices related to TLUD stove acquisition use, as well as quantitative measures of fuel consumption, indoor air quality, and stove usage. 

### Background

**Figure 1:**
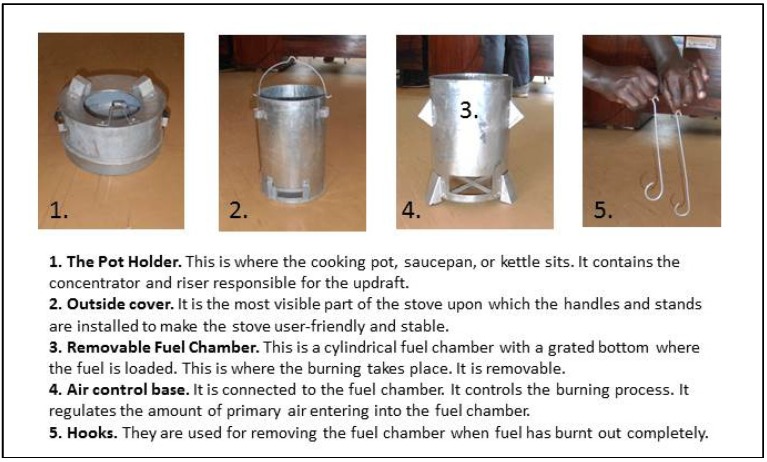
Top-lit updraft (TLUD) gasifier stove.

One fuel-efficient improved cookstove that has shown promise in greatly reducing household air pollution emissions in the laboratory is the top-lit updraft gasifier (TLUD) stove (Figure 1). The TLUD stove has been shown to reduce smoke emissions and efficiently burn biomass [23,24]. While TLUD stove performance has been tested in the laboratory, performance testing in a community setting is needed. As part of this study, fuel consumption, indoor air quality, and stove usage were measured in participating households in Wakiso district to test the performance of TLUD stoves. Those findings will be reported in subsequent publications.

In Kampala, TLUD stoves are locally produced and cost 35,000 Ugandan Shillings, equivalent to nearly US$14. The stove uses small pieces of dry wood or agricultural crop residue as fuel, and in the process of burning biomass can produce charcoal—a unique feature of this stove that allows users to have additional fuel they can burn in a charcoal stove. TLUD stoves, however, require users to change their cooking practices particularly for fuel-loading. There are concerns that the behavioral modifications required of TLUD stoves may limit the ability to scale-up this technology. In order for families to benefit from this improved cookstove, it requires households to both purchase and correctly use the stove over time. Identifying and addressing specific behavior-related barriers and motivators is critical to the successful adoption and correct use of the TLUD stove.

## 2. Methods

### 2.1. Theoretical Framework

There are a number of determining factors that affect acquisition and correct use of an improved cookstove, which may include awareness, availability, cost, and fit with cooking practices. Health-related behaviors, more broadly, are often difficult to change and can be influenced by a variety of personal, financial, social, cultural, and structural factors [24,25]. To increase the adoption and use of improved cookstoves, there is a need to better understand the factors that drive their demand and utilization [19]. 

A behavior change framework is a mechanism to analyze behavior using a specific model or process to facilitate program design, implementation, monitoring, and evaluation. The critical elements of a behavior change strategy include: defining goals and objectives, describing primary and secondary audiences, and understanding behavioral determinants in order to assess appropriate communication channels, interventions, and key messages. There are multiple frameworks, approaches, and models for understanding behavior and behavior change and for designing related interventions [26]. In this study, we utilized a previously developed theoretical framework that draws from several behavioral theories. The opportunity, ability, and motivation framework (Table 1) was adapted from work conducted in the water, sanitation, and hygiene sector [27]. The framework is holistic, drawing upon consumer behavior, social marketing, and health behavior theories and models [27,28]. This is a strength of the framework, as several change theories are needed to address the multiple levels that influence behavior [25].

### 2.2. Study Area and Participants

The study took place in peri-urban areas of Sabagabo sub-county and Kira town council/sub-county in Wakiso District in the Central Region of Uganda. Wakiso district has an estimated population of 1,315,300 (2011) and encircles the capital city Kampala, including peri-urban and rural communities [29]. Literacy is relatively high among both women and men, 74% and 80% respectively [13]. The majority of the population is from the Bantu ethnic group, with a small representation from other Ugandan ethnic groups; the majority speak Luganda. Charcoal and wood are the dominant fuels for cooking among the study population and households typically cook indoors [13]. Qualitative research methods were used to assess current knowledge, attitudes, and practices related to improved cookstoves, focusing on opportunities and barriers for their introduction, acquisition, and use. Each stage of this research and strategy development was guided by the study’s theoretical framework.

**Table 1:** Description of the opportunity, ability, and motivation behavior change framework (reprinted from [27] with permission from Jacqueline Devine).

Behavioral Determinants		
Opportunity	Ability	Motivation
Does the individual have the chance to perform the behavior?	Is the individual capable of performing the behavior?	Does the individual want to perform the behavior?
Access and availability Product attributes Social norms Sanctions and enforcement	Knowledge Skills and self-efficacy Cues for action Social support Roles and decisions Affordability	Attitudes and beliefs Values Perceived susceptibility Behavior attributes Competing priorities Intention Willingness to pay

### 2.3. Design and Data Collection

We utilized purposive sampling to recruit participants for semi-structured interviews and focus group discussions. Focus group discussion participants were selected from healthy adults, ages 18–45, living in Sabagabo or Kira sub-counties, who either had children living at home with them or were village health volunteers at the time of the study. We recruited 24 village health volunteers through the local clinics, and community leaders identified 24 women and 24 men from households to participate in the focus group discussions. Focus group discussion participants were provided with a nominal transport allowance. Semi-structured interviews were conducted with key informants identified by the project team and included health workers, community leaders, government employees from relevant ministries, and staff from nongovernmental organizations (NGO). Interview and discussion guides were developed based on the theoretical framework described above and used open-ended questions and probes to explore potential behavioral determinants. Project staff with extensive qualitative research experience and native fluency in Luganda conducted the interviews and focus group discussions.

In order to collect a variety of perspectives and experiences [30], ten semi-structured interviews and nine focus group discussions were conducted in March 2012. The focus group discussions included: two with mothers, two with fathers, two with mothers and fathers, and three with village health volunteers, all from the Sabagabo and Kira sub-counties. Each focus group discussion had seven or eight people per group and lasted approximately two hours, while the semi-structured interviews lasted approximately 90 min. All interviews were conducted in English and all focus group discussions were conducted in Luganda. The study was approved by the Makerere University, College of Health Sciences, School of Biomedical Sciences Research and Ethics Committee. Study participants verbally consented to participate, which was confirmed by the supervising researcher. 

### 2.4. Data Management and Analysis

Audio recordings were made of all semi-structured interviews and focus group discussions and transcribed. The focus group discussion transcripts were translated into English for the analysis. The interview and discussion transcripts were analyzed thematically by three of the authors using Atlas.ti Version 7, a qualitative research software program (Scientific Software Development GmbH, Berlin, Germany). A hybrid approach that incorporated inductive and deductive methods was used to analyze the data [31]. Initially a template approach to thematic analysis was used, [32] with a codebook developed *a priori* based on the concepts presented in the opportunity, ability, and motivation framework. Once analysis began, additional codes were developed using the general principles of grounded theory [33]. After new codes were identified and agreed upon among the researchers, they were linked to concepts in the theoretical framework. All new codes could be categorized under the overarching themes of opportunity, ability, and motivation, demonstrating the utility of the framework.

## 3. Results and Discussion

### 3.1. Results

Factors relating to the adoption and correct usage of improved cookstoves were identified from the focus group discussion and semi-structured interview transcripts. While most of these findings were similar across the groups, there were some important deviations observed, specifically between parents, village health volunteers, and key informants related to knowledge, perceptions of affordability, and availability of improved cookstoves, as well as differences between mothers and fathers. 

Eight key themes emerged from the data, which were linked to the three overarching determinants presented in the opportunity, ability, and motivation framework. In addition, the findings include determinants relevant to current stove usage, which were important for the design of the behavior change strategy. The TLUD stove was not yet available in Wakiso District at the time of the formative research; therefore, participants’ responses are referencing improved cookstoves in general, unless otherwise noted.

#### 3.1.1. Current Stove Usage

Most respondents reported having between one and three unimproved stoves in their household, with the majority having two (often one made from clay and one traditional three-stone fire). Other types of stoves cited included charcoal stoves, handmade stoves from locally available materials, and non-improved manufactured stoves. Most participants reported that the majority of families in peri-urban areas cook indoors or on semi-enclosed verandas. 

#### 3.1.2. Opportunity: Access to Improved Cookstoves

In regard to availability, most participants reported that the TLUD and other improved cookstoves were not available near their communities. Other types of manufactured (but not improved) stoves were often available for purchase at hardware stores, supermarkets, outdoor markets, and trading centers, although many of the women reported not having seen them for sale, and not knowing where to purchase them. Key informants confirmed that improved cookstoves were not available in many areas. 

The majority of mothers, fathers, and village health volunteers reported they felt the best way to purchase an improved cookstove would be through a reputable, local hardware store to which they could go back and ask questions and get help if they needed it. Most participants agreed that they would feel more comfortable buying a stove from a familiar, permanent shop rather than a door-to-door sales person, who they would not be able to contact if the stove breaks or they have questions. 

*I would also appreciate having a common place sell the cookstoves because when it gets spoilt*, *I can take it back and get a better one for replacement* (*Mother*).

#### 3.1.3. Opportunity: Product Attributes

Mothers and fathers consistently identified durability, affordability, fuel efficiency, speed of cooking, and quick starting as the primary attributes they looked for in a cookstove. Other desirable attributes that were mentioned less frequently included the stove’s ability to keep food warm for a long time, portability and light weight, reduced smoke output, and the ability to cook multiple dishes at the same time. Motivating factors leading to the purchase of a new cookstove in general included: the desire to cook multiple items at the same time; growing family size (requiring preparation of more food); and replacing broken or worn out stoves.

Several village health volunteers and key informants mentioned the reduced risk of fire and burns from improved cookstoves, reduced cooking time, improved children’s and women’s health, and environmental sustainability as important cookstove attributes.

*The good thing with the improved cookstove is that it is durable*, *it keeps energy*, *and it cannot easily* [cause a fire] *in the house* (*village health volunteer*).

…*given the current fuel crisis*, *it*’*s not easy to get fuel even for the working class*….*This charcoal I would have used in a day with the ordinary stove*, *even if I can use it for just two days*. *That is a very big savings*. *Besides that savings*, *they* [mothers and children] *will also realize they no longer have tearing eyes*. *They are not sneezing*; *they are not having sleepless nights because of the soot* (*key informant*).

Attractiveness was not reported to be an important attribute of stoves among parents and village health volunteers, as most participants focused on other factors such as durability and economic benefits. However, some key informants cited attractiveness as an important attribute. One felt that it would be a minor motivation for women. 

*Women also want to be associated with good things*, *so they want something that is nice* (*key informant*).

Mothers, fathers, and village health volunteers mentioned several perceived negative attributes of improved cookstoves in general. These included food cooked on a traditional fire tastes and smells better, lighting improved cookstoves is difficult, high initial cost to purchase the stove, uncertainty of cost savings, difficult to use, may not be hot enough for traditional dishes such as *posho* (a thick maize porridge staple food), and concerns about durability. Other negative attributes mentioned by the parents and volunteers were risks of children getting burned and less heat retention as compared to clay stoves, which were in direct contrast to the perceived positive attributes key informants identified.

#### 3.1.4. Opportunity: Social Norms

Participants (both men and women) felt that if they had neighbors using an improved cookstove, they would be more motivated to purchase one themselves, as they would be able to see how well it worked and how it was used. There was agreement among one focus group discussion with mothers that if women started using the stove, then everyone would start talking about improved cookstoves and increase demand. 

*In most cases*, *the neighbors will come to the home and see how the new stove works and in the process*, *they will get motivated to buy one for themselves* (*mother*).

#### 3.1.5. Ability: Knowledge and Information

Most participants reported knowing what improved cookstoves were, but they were unable to correctly describe them. Often the participants confused improved cookstoves with clay stoves, or charcoal stoves. Only the officials from the ministries of health and energy were familiar with the TLUD stove. Most participants were aware that improved cookstoves use less fuel and produce less smoke than traditional stoves, but they did not necessarily know the specific attributes that differentiate improved cookstoves from more traditional types of stoves. However, some of the key informants had more extensive knowledge of improved cookstoves, and emphasized their environmental and health benefits. A few village health volunteers mentioned hearing radio programming about environmental issues related to cookstoves and the dangers of household air pollution. 

Village health volunteers and key informants reported interest in receiving training on improved cookstoves, as well as enthusiasm to educate community members in order to promote purchase and correct use.

*I would like to know how it is used basically because that*’*s what I hope to tell other people*…*how it is used*, *why it is important*, *of course the benefits*. *How I can promote it so that people accept that it is better than other local stoves*. *I have to convince the community that this is the best stove to use rather than the other local stoves*. *So I have to be trained in this* (*key informant*).

Participants noted that it would be very helpful to attend demonstrations on how to correctly use the TLUD stove, and see how it works in person rather than just hearing about it. Mothers reported that they would feel most comfortable learning about the stoves from peers or women’s groups. Participants suggested that the best way to share information about improved cookstoves was through demonstrations, training community leaders, posters and fliers, integrating messages into existing community sensitization activities, working with health facilities, teaching children about them through the schools, churches, and community radio. Engaging community leaders in the promotion of improved cookstoves was overwhelmingly recommended by all participant groups.

*If a leader is against something you will never sensitize other people because he can even demobilize them not to attend*. *But if you sensitize the leaders first they can even sensitize others* (*key informant*).

#### 3.1.6. Ability: Roles and Decision-Making

Regarding household roles, participants reported that women did most, if not all, of the cooking and meal preparation in the household. A few men said that they helped with tasks such as splitting firewood or cooking when women were not at home. In terms of decision-making, almost all participants reported that women make decisions on what to buy in relation to cooking, but responses were divided on whether women or men do the actual purchasing. Women typically consult with their husbands to explain what they need, and then the husbands often provide the money for the purchase and sometimes do the actual shopping. Many participants stated that the men “don’t care”, “that is not their business”, and they leave all the cooking-related issues to the women. 

*As for me*, *I care less*. *I am more concerned on seeing the food on the table than what type of cookstove my wife uses*. *I think it is up to her to decide* (*father*).

However, a few participants reported that men can be involved in household purchasing decisions. 

*Things have changed these days*; *even husbands see the way their wives are suffering such that they can also purchase stoves* (*village health volunteer*).

#### 3.1.7. Motivation: Perceived Benefits

Women and men indicated that they would readily accept an improved cookstove because of economic considerations, such as fuel efficiency and reduced cooking time. One father explained the financial incentive for the improved cookstove: 

*If a man was buying a sack of charcoal at 60*,*000* [Ugandan Shillings; US$25], *and with these stoves*, *he can buy less charcoal in a week*, *I think he will be supportive in this case*, *hence helping in protecting the environment and saving* [money] *at the same time* (*father*).

Participants identified various perceived benefits of improved cookstoves. The major benefits mentioned by mothers and fathers included money savings and time savings (both from wood collection and reduced cooking time). Other benefits cited by participants included less labor intensive, reduced smoke, heat retention, cleanliness, durability, portability, and reduced environmental degradation. A few individuals also mentioned requiring less firewood, which is often difficult to come by. 

#### 3.1.8. Motivation: Willingness to Pay

Cost was identified as the most important factor in purchasing an improved cookstove. Many participants were concerned about the high cost of the improved cookstove being a prohibitive factor, but others felt that although there was a higher upfront cost, they would experience cost savings over time because of the reduction in fuel costs and improved efficiency. 

*Improved cookstoves are pocket friendly*. *When you are cooking using our traditional stoves*, *you can even spend about 3*,*000 Shillings* [US$1.20] *a day buying that firewood but when you*’*re using that stove of yours* [TLUD] *you can use 1*,*000 Shillings* [US$0.40] *for two days according to what I saw*. *With this stove you save twice* (*key informant*)*.*

However, many respondents reported the tradeoff between the high, one-time, initial cost and the long-term savings was a struggle for many people. Even if a family would save money over time from using an improved cookstove, it would be difficult to pay a large sum at once to purchase the stove. This was highlighted by another key informant: 

*A single deposit*, *at the level of economic strength for the people*, *is such that even 20*,*000* [Shillings; US$8] *is too much for them*. *If somebody is earning like 1*,*000* [Shillings; US$0.40] *per day*, *and needs 20 days to generate 20*,*000 Shillings*. *That is*, *if you exclude eating and other expenses*. *That single deposit is what fails them* (*key informant*).

There was a wide range in what participants felt they would be willing to pay. Most parents agreed that they would not be able to afford more than 5,000–10,000 Ugandan Shillings [US$2–4] for a stove, while village health volunteers reported a willingness to pay slightly more, in the range of 15,000–20,000 Shillings [US$6–8]. 

Responses among mothers, fathers, and village health volunteers were mixed when it came to whether they felt that it would be an incentive to be able to try the stove for a couple of weeks before purchasing. Some felt that this would be a good way to test whether it was worth buying. As one father stated, “I would try it on condition that I will not be forced to buy it.” While others felt suspicious that they would be forced into purchasing the stove after the trial period, or feared that it would break, as pointed out by a village health volunteer: “What if it breaks? Is this a loan?”

#### 3.1.9. Motivation: Perceived Susceptibility to Health Risks

While immediate health benefits, such as less smoke and reduced risk of burns, were acknowledged by many participants, most did not mention health benefits as an important factor when considering the purchase of a new cookstove. When specifically probed about the potential health benefits, mothers from one focus group said that the health benefits would make them more likely to purchase an improved cookstove; fathers from one focus group said it would have no impact on their likelihood to purchase; and both mixed focus groups with fathers and mothers indicated they would be likely to buy if the improved cookstove produced little or no smoke. Even village health volunteers and key informants reported not prioritizing the health issues related to cookstoves.

*For me*, *I was saying that I think we normally do not take time to think about the health benefits of such stoves and therefore we are not sure of the good health side of it* (*village health volunteer*).

*The health risks are not so immediate that you cannot see easily as an ordinary person unless you have an idea*. *Me*, *as an ordinary person and has just been exposed*, *I cannot easily identify health risks* (*key informant*).

Consistent with the finding that participants did not prioritize the health benefits of improved cookstoves, was the widely held perception that traditional three-stone fires must not be dangerous since that is the way people have prepared food for millennia. 

*Our grandparents used the same stoves and they were not affected* [by smoke from traditional fires] (*mother*).

### 3.2. Utilizing Findings to Develop a Behavior Change Strategy

A behavior change strategy development workshop took place over two and a half days in May and June 2012 in Kampala, Uganda. The workshop was facilitated by behavior change and public health experts, and participants included representatives from three government ministries (energy and mineral development, water and environment, and health), NGOs focused on energy conservation and environmental health, village health volunteers, community members, and project partners. The purpose of the workshop was to collaboratively review the formative research findings, and develop a behavior change strategy by prioritizing objectives, identifying motivators and barriers, and suggesting target groups, interventions, messages, and communication channels. The theoretical framework formed the basis for this analysis and strategy development.

A participatory process that utilized Visualization in Participatory Programmes methodology [34] was used to facilitate the workshop. The Visualization in Participatory Programmes process encourages participants to work toward collective agreement [35]. Visualization in Participatory Programmes is based on a combination of different participatory approaches that emphasize visualization, reflection, and engagement, which can be used with participants from varied backgrounds and education levels.

Based on the formative research findings (Table 2), participants identified mothers and fathers in the Kira and Sabagabo sub-counties of Wakiso District as the primary audience and community leaders as the secondary audience. Participants then designed a behavior change strategy through the following steps for each audience:

1. Identifying key behavioral determinants based on the formative research findings and participants’ own expertise.
2. Determining how to strategically respond to each behavioral determinant by describing issues related to the product, price, place, or promotion.
3. Selecting the communication channel and mode of delivery needed to implement this change.
4. Listing which partners or individuals are critical to implement the activity.
5. Drafting key messages to be communicated to the audience, and referencing the particular channel and nature of the behavioral determinant.

The project team used the resulting behavior change strategy to inform the design and implementation of the project. Interventions and messages from the strategy were selected based on community appropriateness, resources available, and messages on stove performance that were accurate. 

**Table 2:** Examples of formative research findings translated into an audience-specific behavior change strategy.

Audience	Formative Research Findings	Interventions	Messages
***OPPORTUNITY***			
**Mothers**	Mothers want to use less fuel.	Use community radio to sensitize women to the key benefits of the TLUD.	TLUD saves money because you use less fuel.
	Mothers want to cook faster.	Demonstrations at community meetings, women’s groups. Brochures with information and illustrations with key benefits, cooking times, and cost savings.	TLUD cooks faster than other cookstoves and saves you time. Two kilograms of *posho* cooks in 10 min.
**Fathers**	Fathers appreciate durability of stoves.	Demonstrations for fathers at bars, sporting events, film halls, and pork joints.	The TLUD is made of 25 gauge iron.
	Fathers like that improved cookstoves save on fuel costs.	Printed brochures with benefits. Demonstrations. Community radio programs.	Use TLUD and get good value for your money in the long run!
**Community Leaders**	People own improved cookstoves to show status.	Testimonials at demonstrations, community events, community radio. Serving food prepared on the TLUD at public events.	Be a role model in your community, use a TLUD stove! Lead the way with a TLUD stove!
***ABILITY***			
**Mothers**	Mothers decide what stoves to buy and fuels to use; they convince their husbands what to purchase.	Demonstrations that encourage wives to talk with their husbands about the benefits that men value most.	Tell your husband the TLUD saves money! In one year, you will save more than 1 million Shillings. You can cook food faster, which means he can eat sooner.
**Fathers**	Fathers are not concerned with food preparation, they only care that it is available when they need it.	Brochures. Demonstrations. Community radio.	The TLUD stove cooks food faster and more efficiently! Your wife can get dinner on the table quicker than before!
**Community Leaders**	Community leaders have high levels of influence in communities.	Demonstrations with mobilized leaders in advance of roll-out with community. Provide t-shirt or other incentive when they buy TLUD.	Buy a TLUD now, and receive a gift to thank you for future advocacy. Your community respects you and follows your lead. Show them how to improve their own lives with a TLUD.
***MOTIVATION***			
**Mothers**	View improved cookstoves as an expensive initial investment.	Work with savings groups to enable women to save money to purchase an improved cookstove.	The TLUD is a good deal for your money. It cooks faster, uses less fuel and produces charcoal. Every household should have a TLUD to make life easier and happier.
**Fathers**	Fathers can be influenced by their neighbors.	Demonstrations. Brochures.	This stove is just hitting the market. Get the latest and greatest before your neighbor!
**Community Leaders**	Leaders recognize the health, financial, and environmental benefits of improved cookstoves.	Demonstrations that emphasize the benefits and how TLUD can improve their community.	Less smoke means less respiratory infections for mothers and children. Use of different fuels will help the environment in the community.

### 3.3. Discussion

While there is a robust body of literature on the health and environmental effects of household air pollution, there is a lack of evidence on the determinants related to the demand for improved cookstoves [36]. In addition, developing and effectively communicating messages to promote improved cookstoves can be a challenge for many organizations [21]. This paper responds to both of these needs by presenting formative research findings on behavioral determinants, and how the findings were used to design a behavior change strategy to promote the acquisition and use of improved cookstoves in Wakiso District, Uganda. Each stage of this process was guided by the opportunity, ability, and motivation theoretical framework, which, based on this experience, appears to be well suited for improved cookstove behavior change interventions. The formative research results revealed participants’ desired attributes for improved cookstoves, as well as the barriers and facilitators for widespread purchase and use. Several important themes, consistent with findings from other studies, emerged that were utilized to design targeted interventions to increase the acquisition and use of TLUD cookstoves. Findings related to the importance of health benefits and the acceptability of a novel sales offer differed from previous studies. 

Although many participants appeared eager for a better alternative to their current stove for a variety of reasons, most were not aware of the options available. Throughout the focus group discussions and semi-structured interviews, it was evident that participants did not know what improved cookstoves were. Although improved cookstoves have been distributed throughout Uganda since the 1980s [11,37,38,39], mothers, fathers, and village health volunteers in the study frequently confused improved cookstoves with clay stoves or any type of stove that was not a three-stone fire. Awareness raising of what makes a stove “improved” is an important step for the successful introduction and use of improved cookstoves [19,38,40], and is central to this project’s activities to promote the TLUD. Information and messages related to the TLUD highlight the differences between other stoves and improved cookstoves, and emphasize the benefits of the latter. 

As found in studies from neighboring countries [41,42], women, as the stove users, appear to be the decision makers for new cookstove purchases. Whether women purchase the stove directly or negotiate with their husband about the purchase varies among households. The behavior change strategy included activities designed to ensure that women have the opportunity to learn experientially about improved cookstoves in social settings that are comfortable and validating, such as integrating messages into demonstrations by peers or in women’s groups and working through other existing community sensitization activities. Sharing ideas with women about how to talk with their husbands about the stove’s benefits are also included.

The most important cookstove attributes that participants reported were all directly related to economic considerations: durability, affordability, fuel efficiency, and starting and cooking times. These attributes are similar to findings from other studies and reviews [19,36], which found that financial and time-savings benefits, rather than health considerations, guide household decision making. The TLUD stove sales materials address these desires and feature those that it can claim, such as fuel efficient and starts and cooks quickly. 

Financial considerations were consistently cited as the most influential factor, both as a barrier and facilitator, affecting motivation. This finding is consistent with other studies of improved cookstoves in East Africa [39,41], as well as findings that socioeconomic status is most correlated with cookstove adoption [19,40]. High initial costs of an improved cookstove was reported as the single most important barrier for purchase, and longer-term savings in fuel and charcoal were cited as the key motivational factor for buying a new stove. Ensuring the low cost of stoves and raising awareness of the future financial benefits of improved cookstoves is part of the behavior change strategy to increase TLUD stove purchases. 

Project implementation priorities have been designed to reflect the primacy of financial considerations. Access to credit has been positively associated with adoption of improved cookstoves [19]. The project has incorporated a savings group approach to address the high initial cost. This approach enables households to pay in installments, with all payments made prior to actual purchase and stove delivery—essentially a group-oriented ‘layaway’ plan. The group-based approach lends itself to information and demonstrations, which were also identified as priorities by participants. A try-before-you-buy sales approach, which was similar to a novel offer found to be effective in another improved cookstove campaign in Uganda [39], did not appear to be compelling to most participants.

Participants reported preferring purchasing a stove from a hardware store or some other static location where they could bring the stove if they experienced problems. In one improved cookstove study, they found that having someone immediately available to address problems with stove use during the first two weeks was essential for continued and correct stove usage [40]. Because this project is introducing a direct sales approach, direct sales agents are trained to provide warrantee information and details on where to take the stove if there are problems with it, as well as where customers can go for help if they have challenges with or questions about using the stove. 

While many participants were aware that improved cookstoves produce less smoke, the potential related health benefits were not reported as an important factor in the decision to buy a new stove. This may be due in part to the health effects related to household air pollution being insidious and often developing over time. It is also possible that it is difficult for participants to imagine health benefits associated with improved cookstoves when they are not used in their communities. This finding that consumers are not motivated by health benefits, is similar to other studies and reviews on improved cookstoves [19,36], and the use of environmental health technologies more broadly [43]. However, this differs from a recent study in rural Kenya, where women cited smoke reduction as a key motivator for using an improved cookstove at baseline [44]. 

The findings from this formative research provided valuable information for the development of a behavior change strategy to increase the adoption and correct use of the TLUD stove. The opportunity, ability, and motivation framework is a useful and appropriate tool for planning formative research, analyzing data, and designing a behavior change strategy. The use of a participatory process that engages key stakeholders to contribute to the development of a behavior change strategy can also be used widely. 

This qualitative research, which consisted of interviews and focus group discussions with a relatively small number of individuals in two sub-counties in peri-urban Uganda, may not be widely generalizable to improved cookstove programs in other contexts. Since this research is formative and improved cookstove use is non-normative in the study areas, it is possible that participants perceptions of benefits will not match their reactions to the stove when it is introduced in their communities. This could limit the appropriateness and ultimately the success of the behavior change strategy. When developing improved cookstove behavior change interventions, it is important to not only understand the factors that influence adoption, but also the determinants of their sustained use [45]. The nature of this formative work prevented the exploration of the factors that influence adoption; however they will be explored in subsequent publications on the study’s outcomes. Even though participants were assured of the anonymity of their responses and that there were no right or wrong answers to the questions, there is the potential for social desirability bias in participants’ responses [46]. Despite the limitations, the formative research methodology; opportunity, ability, and motivation theoretical framework; and participatory behavior change strategy development process are relevant for the design of interventions to improve the acquisition and use of improved cookstoves in general.

## 4. Conclusions

This paper describes how formative research was used to identify behavioral determinants related to the acquisition and use of improved cookstoves in order to design an appropriate behavior change strategy for the TLUD stove in Uganda. The theoretical framework provides a conceptual basis, grounded in social marketing, consumer behavior, and health behavior theories, which can be utilized for designing formative research and developing a behavior change strategy. The findings from the formative research provided valuable information on behavioral determinants that led to a targeted strategy and related interventions. With increasing international support for scaling up improved cookstove use, the approaches and framework presented here can be useful for program planners and researchers interested in identifying behavioral determinants and designing and evaluating behavior change interventions to increase the use of improved cookstoves. 
